# Determinants of measles vaccination dropout among 12 − 23 months aged children in pastoralist community of Afar, Ethiopia

**DOI:** 10.1186/s12879-022-07350-1

**Published:** 2022-04-14

**Authors:** Chekole Hailu, Girmatsion Fisseha, Aregawi Gebreyesus

**Affiliations:** 1 Afar Regional Health bureau, Afar, Ethiopia; 2 School of Public Health, College of Health Sciences, Mekelle University, P. O. Box 1871, Tigray Mekelle, Ethiopia

**Keywords:** Measles vaccine, Dropout rate, Determinates, Pastoralist community, Ethiopia

## Abstract

**Background:**

Measles is a viral disease and a leading vaccine-preventable childhood killer. More than 95% of measles deaths occur in countries with low incomes and weak health infrastructures. In response to this, Ethiopia prepared a measles elimination strategic plan to achieve by 2020. However, based on the Mini-Ethiopian demographic health survey 2019 the full coverage of immunization is 43% at the country level and it is lowest (20%) in the Afar region where this study was conducted. Therefore, this study aimed to identify the determinants of the measles vaccine dropout rate in Afar regional state which is one of the pastoralist communities in Ethiopia.

**Methods:**

Community based unmatched case-control study design was used. The study was conducted in Awash district of Afar regional state, Ethiopia from June 1st -30th 2018. Data were collected from a study unit of 12–23 months old children. For this study, a sample of 166 cases and 331controls were selected by simple random sampling methods and the total sample size was 497. Data were collected using a pretested structured questionnaire by health workers using the local language. Data were entered into Epi-info − 7 and analyzed by SPSS version 20 software and logistic regression was used to assess the determinants measles dropout rate.

**Results:**

A total of 487 children participated in this study with a response rate of 97.9%. More than half of the children were female (53.3%) and 113 (35.2%) children mothers’ were not attended formal education. Mother who had antenatal care ≤ 2 visits [AOR:=5.7(3.2–10.14)], being in the birth order of 1 − 3 [AOR = 4.47(1.63–12.29)], long waiting time > 60 min at nearby health facility for vaccine [AOR = 2.37(1.36–4.15)], households visit by health extension workers [AOR = 2.03(1.12–3.66)], pregnant women not participating with women development army [AOR = 3.5(1.94–6.18)], and poor maternal knowledge on vaccination [AOR = 3.30(1.9–5.73)] were significant determinants with measles vaccination dropout rate.

**Conclusions:**

Health facility and mother characteristics were the determinants of the measles vaccine dropout rate. Therefore, tracing and strict follow up by the health extension works using home visits and women development army at the pastoralist community is necessary to reach them.

## Background

Measles is an acute, highly communicable serious respiratory viral disease characterized by fever, maculopapular erythematous rash, cough or coryza or conjunctivitis [1]. Infection can be transmitted four days before the onset of rash and four days after the eruption of rash. Almost all (100%) unimmunized population will become infected [2, 3]. Immunization is the cheapest public health intervention available in the world. Even though nearly 30 million children born every year in developing countries are not fully immunized [4, 5]. In Ethiopia, the measles vaccination (MCV) is given at nine months after birth for all children under the government mandate of the subsidized program [6].

Measles can cause serious illness, lifelong complications, and death and it was estimated to 15,000 to 60,000 cases of blindness annually globally [1]. More than 95% of measles deaths and morbidities occur in countries with low incomes and weak health infrastructures. It is very important to complete all the immunizations to protect children from all the Expanded Program of Immunization (EPI) target diseases [4, 7]. It is important to understand that the status of disease elimination can be achieved only if the vaccine reaches every child globally. To eliminate measles by 2020, WHO African Region (AFR) countries and partners planned to achieve ≥ 95% MCV2 coverage; improving supplemental immunization activities (SIA) quality, and by using readiness, intra-SIA and post SIA assessment tools; implementing fully elimination-standard surveillance; conducting annual district-level risk assessments; and establishing regional and national verification commissions for elimination [4, 8]. Ethiopia also prepared a measles elimination strategy aiming to achieve measles elimination by 2020. However, based on the mini-Ethiopian demographic health survey (EDHS) 2019 report, the coverage of measles immunization is 59% at the country level and it is lowest in the Afar region that is 28.5% [1, 6–8].

Different studies are conducted on identifying determinants of full coverage of immunization. According to those literatures, there are socio-demographic determinants like age of the mothers /caregiver, sex mothers /caregiver, ethnicity of the respondent, educational status of mothers /caregiver, marital status, religion status, residence area, occupational status, family size of the respondents, and monthly income of the/mother/ caregiver [6, 8–15]. Health facility determinant**s** such as waiting time at the health facility, distance to the health facility, visiting households by Health Extension Workers (HEWs), convenient working time for vaccination, vaccinator absents, and schedule of the vaccine [6, 9, 10, 13, 15–26]. Maternal (caretaker) determinants like antenatal care (ANC) follow up, knowledge about immunization, tetanus toxoid < 2 dose vaccination, delivery, PNC (postnatal care) services, having more children, and participation on WHDAs [9, 10, 26–29, 13, 15, 18, 20–24].

Based on the Afar regional health bureau annual report, the dropout rate of the measles vaccine was > 25% [6]. To achieve the full immunization coverage, dropout rates should be decreased. Dropout rates are used to measure program continuity of children in immunization and dropout rates greater than 10% usually indicate a serious quality problem and need to be addressed [7, 12, 30]. Unlike the studies in Ethiopia, this study was carried out in a pastoralist society to assess the determinants of measles vaccine dropout status due to the high measles dropout rate in the area. Therefore, this study aimed to identify the determinants of the measles vaccine dropout in the Awash district of Afar regional state, Ethiopia 2018.

## Methods

### Study setting and design

This study was conducted using an unmatched case-control community-based study design from June 1st to 30th 2018 in Awash district of Afar regional state. The Awash district is located in the north-east part of the country in the Great Rift Valley and 365 km far from Semera capital of the Afar region or 230 km far from Addis Ababa capital city of Ethiopia. The district had one public health center and 10 private clinics. The district had a total population of 17,337 based on the 2007 census population projection.

### Study population

The study population of this study was children 12 to 23 months old who were living in the Awash district of the Afar region. Children 12–23 months old who are vaccinated for the first dose of Pentavalent vaccine (DPT-HepB-Hib) and drop-out for measles vaccination of the town were included in the study as a case group. However, children who didn’t vaccinate for the first dose of pentavalent vaccine (DPT-HepB-Hib) were excluded from the study. Whereas, children 12–23 months old who are vaccinated for the first dose of Pentavalent vaccine (DPT-HepB-Hib) and completed for measles vaccination were included in the study as a control group. However, children 12–23 months old who didn’t get the first dose of the pentavalent vaccine (DPT-HepB-Hib) of the town were excluded.

### Sample size and procedure

The sample size calculation was calculated using determinants of measles vaccination dropout in different studies done on the previous time. Sampling was determined based on the double proportion formula on the software of Epi Info StatCalc version 7 after considering the following assumptions; 95% confidence interval (CI), 80% power, 1:2 ratio of cases to controls group, odds ratio (OR) of 1.79 and taking the proportion of 44.1%, Evidence of vaccination as a determinant for measles vaccination dropout (from a population-based case-control study on Predictors of defaulting from completion of child immunization in south Ethiopia) [9]. And we added an expected non-response rate of 10%. Finally, the total sample size was 497 (166 cases and 331 controls) with the ratio of 1:2 case to control.

The sampling procedure was based on simple random sampling procedures with random table generation to allocate the appropriate study subjects. We identified the cases and controls at the community level using the Awash health center immunization book and vaccination card for verification before actual data collection. Then we took our sampling population using a simple random sampling method from the randomly selected *kebelles (*Kebelle is the smallest administrative place in Ethiopia).

### Measurements

The dependent variable for this study was the measles vaccination dropout status. The independent variables were socio-demographic characteristics (such as the age of the mothers and child, sex of the child, ethnicity, educational status of mothers, health facility determinants (such as waiting time at a health facility, distance to the health facility, convenient working time for vaccination, and schedule of the vaccine).

Measles dropout: measures at which children who started the vaccination with Pentavalent 1 vaccine (DPT-HepB-Hib) but failed to receive the measles vaccine, hence failing to complete the schedule [32].

Low dropout rate: Dropout less than 10% of measles vaccination doses [30].

High dropout rate: Dropout greater than 10% of measles vaccination doses [30].

Long waiting time: Mothers who had vaccinated their children waiting time at the facility were greater than or equal to 60 min during the immunization sessions.

Good maternal knowledge: Mothers knowledge whose response rate scored with the mean value greater than five points from the total ten knowledge questions.

Inconvenient working hours: Mothers who had vaccinated their children not comfortable for facility working hours during the immunization sessions.

### Data collection tool and technique

A validated tool was adapted from reviewed of different relevant literatures [9, 13–15, 20, 22, 23, 25, 31, 33]. The questionnaire translated into the local language before the data collection by two experts who were speakers of English and the local language independently. After the data collection also it was re-translated back to English. The questionnaire has five main parts which include socio-demographic, maternal/caregiver/reproductive health status, and child measles vaccine status, knowledge, and health facility-related questions. Data was collected through direct face to face interviews of the mothers/ caregivers of the children by four trained data collectors with two supervisors using the prepared and pretested structured questionnaire. The data collection process started by identified both cases and control groups using a vaccination card and Awash health center record book of immunization. The cases and controls group were identified (case those who dropout measles vaccine) and (control those who completed the measles vaccine). The data collection process was done by checking evidence of vaccination cards and Awash health center record book with compilations certificate in addition to oral response mothers /caregiver / and other evidence that shown the child vaccinated for Pentavalent vaccine (DPT-HepB-Hib) one and measles or drop out for measles.

### Data analysis

Data were cleaned and coded for completeness and consistency and processed and analyzed using SPSS software version 20. Descriptive statistics were used to explain the baseline characteristics of the study subjects. Descriptive statistical analysis such as simple frequencies, measures of central tendency and measures of variability were used to describe participant characteristics. Then results were presented using frequencies, summary measures, tables, and charts.

All independent variables were found statistically significant in chi-square (X2) tabulation/bivariate analysis at the p-value of ≤ 0.25 considered for multivariable logistic regression analysis. After the above variables (which were statistically significant in chi-square tabulation/bivariate analysis at the P-value of < 0.25) entered into the multivariable logistic regression analysis, significant determinants were identified at the p-value of ≤ 0.05 and reported as a determinant. Good fitness also checked using Hosmer-Lemeshow statically significance of > 0.05.

### Ethical consideration

Ethical clearance was approved by the Mekelle University, College of Health Science, and Institutional Health Research Ethics Review Committee (IHRERC). A permission letter was also obtained from the Afar regional state health officials. Moreover, informed written consent was obtained from each participant after explaining the purpose and benefits of the study (the age of all participant mothers was ≥ 16 years old). Confidentiality was kept by using a code number in which was immediately detached and filed separately in a confidential manner.

## Results

### Characteristics of mothers/caregivers

A total of the 487 mothers/caregiver with the age of 12–23 months of children (166 cases and 321 controls) participated in the study making a response rate of 97.9%. The mean ± SD age of the respondents was 26.9 ± 5.3for cases and 26 ± 4.5 years for the control group (Table 1).

**Table 1:** Socio-demographic characteristics of the children and family on determinants of measles vaccination dropout among 12–23 months children in Awash district Afar Regional State, Ethiopia 2018.(N = 487)

Variables	Category	Case, n = 166 (100%)	Control, n = 321 (100%)
Maternal age	≤ 20 years 20–29 years 30–39 years ≥ 40 years	22 (13.3) 28 (16.9) 66 (39.8) 50 (30.0)	50 (15.6) 58 (18.1) 135 (42.1) 78 (24.3)
Child age	12–18 months 19–23 Months	101(60.8) 65 (39.2)	174 (54.2) 147 (45.8)
Child sex	Male Female	77 (46.4) 89 (53.6)	150 (46.7) 171 (53.3)
Religious	Muslim Christian	128 (77.1) 38 (22.9)	215 (67.0) 106 (33.0)
Ethnicity	Algona Afar Oromo Amhara Tigrian Wolaita Others *	44 (26.5) 35 (21.1) 35 (21.1) 27 (16.3) 3 (1.8) 17 (10.2) 5 (3.0)	102 (31.8) 70 (21.8) 55 (17.1) 53 (16.5) 14 (4.4) 12 (3.7) 15 (4.7)
Mother education	No Formal education Formal education	80 (48.2) 86 (51.8)	113 (35.2) 208 (64.8)
Mother occupation	Housewife Employed	123 (74.1) 43 (25.9 )	237 (73.8) 84 (26.2)
Marital status	Unmarried Others Married	18 (10.8) 148 (89.2)	14 (4.4) 307 (95.6)
Father education	No Formal education Formal education	94 (56.6) 72 (43.4)	146 (45.5) 175 (54.5)
Father occupation	Unemployed Employed	25 (15.1) 141 (84.9)	17 (5.3) 304 (94.7)
Family size	≤ Five > Five	118 (71.1) 48 (28.9)	251(78.2) 70 (21.8)
House Hold income	≤ 1500 ETB > 1500 ETB	43 (25.9) 123 (74.1)	49 (15.3) 272 (84.7)

*Gurage, Somali and Sidamo

Mothers who had good knowledge of immunization were 216(44.4%) (Table 2).

**Table 2:** Knowledge characteristics on measles vaccination dropout among 12–23 months children in Awash district Afar Regional State, Ethiopia 2018. (N=487)

Variables (Mothers/caregiver/Knowledge	Category	Cases, n = 166 (%)	Control, n = 321 (%)
Knowledge of measles diseases	Yes	64 (38.6)	235 (73.2 )
	No	102 (61.4)	86 (26.8)
Knowledge of measles vaccine	Yes	74 (44.6)	234 (72.9)
	No	92 (55.4)	87 (27.1)
About Pentavalent-1 Vaccination schedule	Yes	134 (80.7)	309 (95.5)
	No	32 (19.3)	16 (5.0)
Measles-Vaccination giving age	Yes	111 (66.9)	271 (84.4)
	No	55 (33.1)	50 (15.6)
Measles-Vaccine Prevent measles disease	Yes	196 (61.1)	65 (39.2)
	No	125 (38.1)	101 (60.8)
Facility Measles-Vaccine schedule/week	Yes	252 (78.5)	98 (59.0)
	No	69 (21.5)	68 (41.0)
Immunization Key message-1 (Benefit of Vaccine)	Yes	171 (53.3)	31 (18.75)
	No	150 (46.7)	135 (81.3)
Immunization Key message-2 (AEFI )	Yes	192 (61.4)	102 (61.4)
	No	124 (38.6)	64 (38.6)
Immunization Key message-3(vaccine card handling)	Yes	259 (80.7)	105 (63.3)
	No	62 (19.3)	61 (36.7)
Immunization Key message -4Appointment	Yes	286 (89.1)	124 (74.7)
	NO	35 (10.9)	42 (25.3)

About 133(80.5%) from cases and 286 (89.1%) control group women gave birth in health facilities. Mothers who visited health facility were attended antenatal care follow up less than or equal two visited 128 (77.1%) cases and 244 (76.0%) controls group and mothers had tetanus toxoid vaccinated 120 (72.3%) cases, 203 (63.2%) control group were during their pregnancy time (Table 3).

**Table 3:** Reproductive health characteristics of mothers on measles vaccination dropout among 12–23 months children in Awash district Afar Regional State, 2018. (N=487)

Variables	Category	Case,	Control,
		n=166(%)	n=321(%)
Facility birth attended mothers	Yes	133 (80.1)	286 (89.1)
	No	33 (19.9)	35 (10.9)
Postnatal care attended mothers	Yes	39 (23.5)	128 (39.9)
	No	127 (76.5)	193 (60.1)
Pregnant women participated in women's development army	Yes	58 (34.9)	153 (47.7)
	No	108 (65.1)	168 (52.3)
Number of Parity	≤ 2 parity	115 (69.3)	244 (76.0)
	> 2 parity	51(30.7)	77 (24.0)
An antenatal care follow-up visit	≤ 2 ANC	128 (77.1)	129 (40.2)
	>2 ANC	38 (22.9)	192 (59.8)
Mothers tetanus toxoid vaccinated	≤ 2 TT	120 (72.3)	203 (63.2)
	>2 TT	46 (27.7)	118 (36.8)

### Reasons for measles vaccine dropout

**Fig. 1:**
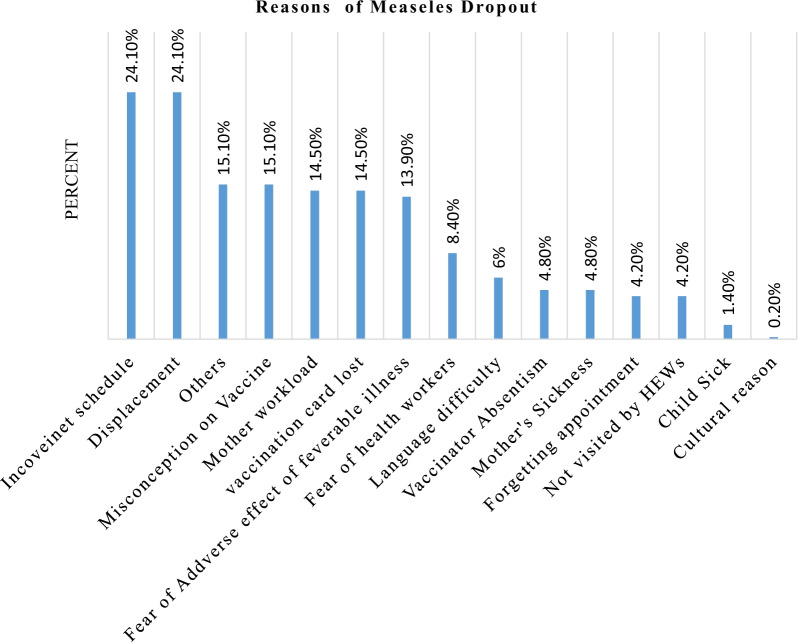
Reasons for measles vaccination dropout among children aged 12–23 months in Awash district of Afar regional state, Ethiopia 2018

This finding showed that reasons for measles vaccine dropout; inconvenient schedule and displacement were the highest reason (24.10%) followed by miss understanding about immunization and other reasons and the lowest insignificant reasons were cultural (0.2%) as shown (Figure 1).

### Determinants of measles vaccination dropout rate

All independent variables were found statistically significant in chi-square (X2) tabulation/bivariate analysis at the p-value of ≤ 0.25 considered for multivariate regression analysis. After the above variables (which were statistically significant in chi-square tabulation / bivariate analysis at the P-value of < 0.25) entered into the multivariate regression analysis, significant determinants were identified at the p-value of ≤ 0.05.

Children who were born from mothers who had less than or equal to two antenatal care follow up visits during their pregnancy period were five times more likely to dropped out measles vaccine compared to those children who were born from mothers who had more than or equal to two antenatal care follow up visit [AOR (95%CI) = 5.7 (3.2-10.14)].

Children who were born from mothers who had birth orders one to three were four times more likely to drop out of the measles vaccine compared to those children who were born from mothers without/no birth order [AOR (95% CI) = 4.47(1.63–12.29)].

Children who were born from mothers who had visit health facility for immunization session waited greater than sixty minutes were two times more likely dropped out measles vaccine compared to those children who were born from mothers waited for less than sixty minutes at health facility during the immunization session [AOR (95% CI) = 2.37(1.36–4.15)]. The odds of measles vaccine dropout rate among children who were born from mothers who’s households visited by health extension worker more than one month was two times [AOR (95% CI) = 2.03 (1.12–3.66)] compared to those children who were born from mothers who’s households visited by health extension worker less than one months.

Mothers who had poor knowledge about immunization were more likely to drop out of their children’s measles vaccination compared to those having good knowledge of immunization [AOR (95% CI) = 3.30 (1.9–5.73)]. Children who were born from mothers those didn’t have participation meeting with women development army were more likely to dropped out measles vaccine compared to children who were born from mothers those had participation meeting with women development army during their pregnancy period [AOR (95% CI) = 3.5 (1.94–6.18)] (Table 4).

**Table 4:** Multivariate analysis of determinants of measles vaccine dropout among 12–23 months old children in Awash district Afar Regional State, Ethiopia 2018

Variables	Cases n = 166 (%)	Controls n = 331(%)	COR (95% CI)	AOR (95% CI)
Religious status				
Christian	38 (22.9)	106 (33.0)	1	1
Muslim	128 (77.1)	215 (67.0)	1.66 (1.08–2.55)	1.68 (0.895 − 3.17)
Marital status				
Married	148 (89.2)	307 (95.6)	1	1
Unmarried	18 (10.8)	14 (4.4)	2.667 (1.291–5.5)	0.784 (0.203 − 3.03)
Maternal education				
Formal	86 (51.6)	208 (64.8)	1	
No formal	80 (48.2)	113 (35.2)	1.712 (1.179–2.5)	1.68 (0.945 − 2.99)
Husband education				
Formal	72 (43.4)	175 (54.5)	1	1
No formal	94 (56.6)	146 (45.5)	1.565 (1.07–2.28)	0.717 (0.417 − 1.23)
Husband occupation				
Employed	141 (84.9)	304 (94.7)	1	1
Unemployed	25 (15.1)	17 (5.3)	3.17 (1.659–6.06 )	2.44 (0.889 − 6.69)
Household income				
>1500 ETB	123 (74.1)	272 (84.7)	1	1
≤1500 ETB	43 (25.9)	49 (15.3)	1.941 (1.22–3.08)	1.99 (0.907–4.365)
HH visited by HEWs				
≤ 1 month	33 (19.9)	131 (40.8)	1	1
>1 month	133 (80.1)	190 (59.2)	2.779(1.79–4.3)	2.03(1.12–3.66)*
Facility waiting time				
≤ 60 min	40 (24.1)	163 (50.8)	1	1
>60 min	126 (75.9)	158 (49.2)	3.2496(2.14–4.9)	2.37(1.36–4.15)*
Birth order				
No birth order	9(5.4)	62 (19.3)	1	1
Birth order1–3	157 (94.6)	259 (80.7)	4.18(2.019–8.64)	4.47 (1.63–12.29)*
Maternal knowledge				
Good	48(28.9)	226(70.4)	1	1
Poor	118(71.1)	95(29.6)	5.848 (3.9–8.8)	3.30 (1.9–5.73)*
N^o^_ of Antenatal care				
> 2 Visit	38 (22.9 )	192 (59.8)	1	1
≤ 2 Visit	128 (77.1)	129 (40.2)	5.01 (3.28–7.67)	5.7 (3.2–10.14)*
Participation of pregnant women with WD army				
YES	58 (28.9)	153(47.7)	1	1
NO	108 (71.1)	168 (52.3)	1.696 (1.15–2.5)	3.5 (1.94–6.18)*
Facility birth				
YES	133 (80.1)	286 (89.1)	1	
NO	33 (19.9)	35(10.9)	2.027(1.208–3.4)	1.084 (0.511–2.301)
Postnatal care follow up				
YES	39 (23.5)	128 (39.9)	1	
NO	127 (76.5)	193 (60.1)	2.1597 (1.42–3.3)	1.762 (0.970–3.202)
Good well coming at the facility registration unit				
YES	48 (28.9)	217 (67.6)	1	1
NO	118 (71.1)	104 (32.4)	5.129 (3.408–7.7)	3.01 (1.7–5.27)
Convenient working hrs				
YES	88 (53.0)	279 (86.9)	1	1
NO	78 (47.0)	42 (13.1)	5.888 (3.8–9.19)	3.1 (1.73–5.63)
Possession of child vaccine card				
YES	121 (72.1)	282 (87.1)	1	1
NO	45 (27.1)	39 (12.1)	2.69 (1.7–4.3)	3.33 (1.65–6.7)

*Significant variables (p-value < 0.05)

## Discussion

The findings of this study showed that mothers who had Antenatal care ≤ 2, being in the birth order of 1 − 3, long waiting time > 60 min, households visit by health extension workers, pregnant women not participating with women development army and poor maternal knowledge were determinants of measles vaccination dropout.

Regarding this study, households didn’t visit by health extension worker for more than one month is two times more likely significantly associated with measles vaccine dropout. This finding is consistent with a study conducted in Tigray [15]. This may be the health extension workers increases the knowledge and awareness about the importance of vaccination.

In this study, long waiting time at the health facility was three times more likely significantly associated with measles vaccine dropout. This finding was consistent with a study conducted in Sinana, district of southern Ethiopia [33]. This might, long waiting time causes the dissatisfaction of clients and results for vaccination dropout. Being birth order one to three was another determinant that contributed to the measles vaccine dropout. This finding is similar to a study conducted in Hawassa, Arbegona district, and India [20, 25, 29]. As the number of children in the family increases, family resources, including attention and time is shared among the children. This could result in children born late in the family not getting the full vaccine.

Regarding this study, poor maternal knowledge is three times more likely associated with measles vaccine dropout. This is consistent with studies conducted across the globe [9, 15, 17, 20, 23, 26, 27, 29, 31, 33]. It could due to mothers not getting enough knowledge and awareness about immunization, and the knowledge may help them to have an interest and vaccinate their child.

Based on our study, antenatal care follow up visits less than or equal to two visited mothers were five times more likely to be dropout for the measles vaccine. This result is similar with different studies carried out in different countries [13, 14, 16, 17, 22, 24, 27, 33]. This might increase the exposure and positive attitude towards the vaccination of their children.

Children who were born from mothers those didn’t have participation meeting with women development army were more likely to dropped out measles vaccine compared to children who were born from mothers those had participation meeting with women development army during their pregnancy period. This may be the woman who provides full awareness about vaccination importance. This finding is consistent with a study conducted by Laelay Adiabo, Tigray Ethiopia [15].

### Strengths and limitations of the study

This study was done in the pastoral community at the community level and this may give a new perspective towards the pastoral society and may have external validity for other pastoral societies of the globe. However as a limitation of this study; since the study was done in the pastoral community there might be a movement of individuals to other residencies within the district and to minimize this problem and recall bias we used the health facility immunization registration book, vaccination certificate and Health Extension workers of the district.

## Conclusions

The findings of this study showed that mother who had Antenatal care ≤ 2, being in the birth order of 1 − 3, long waiting time > 60 min, households visit by health extension workers, pregnant women not participating with women development army and poor maternal knowledge were the determinates of measles vaccination dropout. Ethiopia has already prepared the measles elimination strategy by 2020 in all parts of the country. Therefore, tracing and strict follow up by the health extension works using home visits and women development army at the pastoralist community is necessary to achieve its elimination policy.

## Data Availability

All the data supporting the findings is contained within the manuscript, when there is in need the data-set used for the present study’s conclusion can be accessible from the corresponding author on reasonable request.

## Abbreviations

MCV: Measles vaccination
WHDA: Women health development army
ANC: Antenatal care
HEW: Health extension worker
DPT-HepB-Hib: Diphtheria, Pertussis, Tetanus, Hepatitis B, Homophiles influenza type B
WHO: World Health Organization
ARHB: Afar Regional Health Bureau
SPSS: Statistical package for social science
AOR: Adjusted odds ratio
CI: Confidence interval
